# Experimental quantum teleportation of propagating microwaves

**DOI:** 10.1126/sciadv.abk0891

**Published:** 2021-12-22

**Authors:** Kirill G. Fedorov, Michael Renger, Stefan Pogorzalek, Roberto Di Candia, Qiming Chen, Yuki Nojiri, Kunihiro Inomata, Yasunobu Nakamura, Matti Partanen, Achim Marx, Rudolf Gross, Frank Deppe

**Affiliations:** 1 Walther-Meißner-Institut, Bayerische Akademie der Wissenschaften, 85748 Garching, Germany.; 2 Physik-Department, Technische Universität München, 85748 Garching, Germany.; 3 Department of Communications and Networking, Aalto University, 02150 Espoo, Finland.; 4 RIKEN Center for Quantum Computing (RQC), Wako, Saitama 351-0198, Japan.; 5 National Institute of Advanced Industrial Science and Technology (AIST), 1-1-1 Umezono, Tsukuba, Ibaraki 305-8568, Japan.; 6 Research Center for Advanced Science and Technology (RCAST), The University of Tokyo, Meguro-ku, Tokyo 153-8904, Japan.; 7 Munich Center for Quantum Science and Technology (MCQST), Schellingstr. 4, 80799 Munich, Germany.

## Abstract

The field of quantum communication promises to provide efficient and unconditionally secure ways to exchange information, particularly, in the form of quantum states. Meanwhile, recent breakthroughs in quantum computation with superconducting circuits trigger a demand for quantum communication channels between spatially separated superconducting processors operating at microwave frequencies. In pursuit of this goal, we demonstrate the unconditional quantum teleportation of propagating coherent microwave states by exploiting two-mode squeezing and analog feedforward over a macroscopic distance of *d* = 0.42 m. We achieve a teleportation fidelity of *F* = 0.689 ± 0.004, exceeding the asymptotic no-cloning threshold. Thus, the quantum nature of the teleported states is preserved, opening the avenue toward unconditional security in microwave quantum communication.

## Teaser

Experimental microwave teleportation brings quantum local area networks within reach by exceeding the no-cloning limit.

## INTRODUCTION

The modern field of quantum communication thrives on the promise to deliver efficient and unconditionally secure ways to exchange information by exploiting quantum laws of physics. Here, quantum teleportation (QT) stands out as an exemplary protocol, allowing for the disembodied and safe transfer of unknown quantum states using quantum entanglement and classical communication as resources (*1*). The experimental feasibility of QT with propagating waves, relevant to communication scenarios, has been demonstrated at optical frequencies (*2*–*4*). However, an implementation with propagating microwaves has been missing so far. Meanwhile, the recent progress in quantum computation with superconducting circuits (*5*) led to the requirement of quantum communication between spatially separated superconducting processors operating at microwave frequencies. One way to achieve this communication task would be to use propagating two-mode squeezed (TMS) microwaves to entangle remote qubits (*6*, *7*) or to teleport microwave states to interface between remote superconducting systems. Here, we demonstrate the deterministic QT of coherent microwave states by exploiting two-mode squeezing and analog feedforward over a macroscopic distance of *d* = 0.42 m. We achieve teleportation fidelities *F* = 0.689 ± 0.004 exceeding the asymptotic no-cloning *F*_nc_ = 2/3 threshold (*8*, *9*). Our results provide a key ingredient for future microwave quantum local area networks and modular quantum computing (*10*).

QT allows one to achieve the classically impossible goal of transferring an unknown quantum state from one place to another without directly sending it. This task is usually quantified with a teleportation fidelity *F*, which expresses the overlap in the phase space between an unknown input state and a teleported output state. In terms of the corresponding density matrices ρ_in_ and ρ_out_, the QT fidelity can be expressed as $F={\left(\text{Tr}\sqrt{\sqrt{\rho_{\text{in}}}\rho_{\text{out}}\sqrt{\rho_{\text{in}}}}\right)}^{2}$. In this case, a transition to the quantum realm occurs when exceeding the classical fidelity threshold *F*_ct_. This situation is only possible by using nonclassical correlations such as quantum entanglement. The exact value of *F*_ct_ is a subject of many scientific discussions (*8*). It depends on the set of teleported states and respective Hilbert space dimension. In the case of continuous-variable propagating fields, *F*_ct_ = 0 for arbitrary input states, while for the particular task of teleporting coherent states, one finds *F*_ct_ = 1/2 (*9*). For qubit states, this threshold is different, namely, *F*_ct, qubit_ = 2/3, and has been experimentally overcome with superconducting qubits (*11*, *12*). These threshold values are connected with a violation of the Clauser-Horne-Shimony-Holt inequality that expresses the fact that nature cannot be described by local hidden-variable theories (*8*, *13*). In this context, teleportation of continuous-variable Gaussian states has several important technological advantages over discrete-variable states: Experimental generation and control of weak coherent tones are significantly easier as compared to Fock states, because the former can be achieved with conventional microwave generators. Respectively, continuous-variable entangled states (in the form of TMS light) can be generated in the steady-state regime by using weakly nonlinear superconducting devices, such as various Josephson parametric devices. This steady-state approach allows for deterministic entanglement generation that results in higher communication bit rates, as compared to frequently used nondeterministic entanglement generation schemes with discrete variables.

## RESULTS

### Protocol and experimental setup

**Fig. 1.**
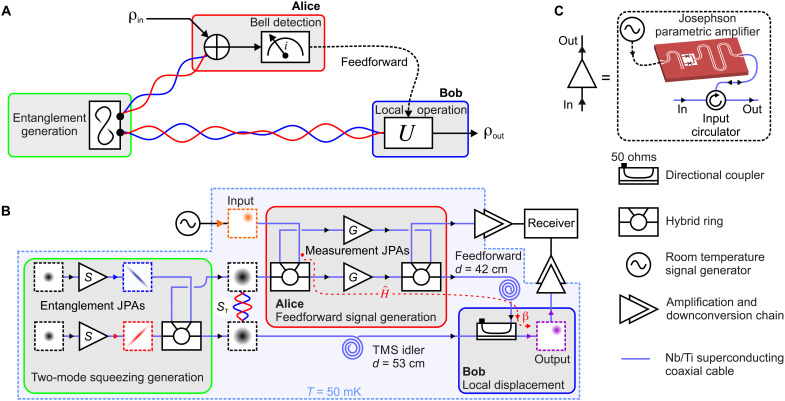
QT of propagating microwaves: concept and implementation. (**A**) General concept. (**B**) Our experimental implementation of QT with propagating quantum microwaves and analog feedforward (also see note S1 for the full technical schematics). Here, an unknown input coherent state is teleported from Alice to Bob by exploiting quantum entanglement characterized by the two-mode squeezing level *S*_T_ ≲ *S*. The feedforward signal is generated by the measurement JPAs with the degenerate gain *G*, in combination with two hybrid rings and a local displacement operation on Bob’s side. The latter is implemented with a directional coupler with the coupling β = −15 dB. Plots in dashed boxes represent quantum states in the quasi-probability Wigner phase space spanned by field quadratures *p* and *q*. Red dashed line marks a particular input signal path corresponding to operator $\hat{H}$. (**C**) Details and labels of various experimental elements.

A general QT protocol consists of several fundamental steps (also see Fig. 1A): (i) entanglement generation and distribution between communication parties (usually denoted as Alice and Bob), (ii) local operations on Alice’s side aiming at generation of a suitable feedforward signal, and (iii) feedforward and a local unitary operation on Bob’s side. The latter operation results in teleportation of the unknown input state by combining the feedforward signal with the entangled resource state (*14*).

Our experimental implementation of QT with propagating microwaves relies on superconducting flux-driven Josephson parametric amplifiers (JPAs) for generation and manipulation of entangled TMS microwave states (*15*–*18*). To describe the continuous-variable states of propagating microwave fields, it is convenient to introduce a pair of conjugate operators—the quadratures $\hat{p}$ and $\hat{q}$. They are analogous to the canonically conjugate variables of position and momentum of a massive particle. A scheme of our QT setup is shown in Fig. 1B. Here, we use two entanglement JPAs in combination with a hybrid ring (microwave beam splitter) for generation of path-entangled TMS states ∣ψ_TMS_〉 at the outputs of the hybrid ring (*16*). These JPAs emit microwave states that are squeezed along different quadratures with the squeezing level *S* below vacuum fluctuations. When superimposed at the hybrid ring, these states produce outputs that, locally, look like classical thermal noise. Nevertheless, they have strong quantum correlations between field quadratures in different propagation paths. These correlations can be described by the two-mode squeezing level *S*_T_, which is, in our case, well approximated by the local squeezing *S* ≳ *S*_T_ (see Materials and Methods). In the limit of infinite squeezing *S* → ∞, one obtains perfect correlations, e.g., $\left({\hat{p}}_{1}+{\hat{p}}_{2})|\psi_{\text{TMS}}\rangle \to \delta (p_{1}+p_{2}\right)$, in the case of anticorrelated *p* quadratures.

### Entanglement distribution and feedforward

Owing to the propagating nature of our TMS states, we can straightforwardly distribute the entangled states between Alice and Bob via superconducting niobium-titanium coaxial cables with characteristic losses as low as 10^−3^ dB/m at frequencies *f* ≃ 5 GHz, thus implementing step (i) of the QT protocol. We extract the characteristic losses of superconducting NbTi cables from independent resonator quality factor measurements at the ambient temperature of *T* = 1.6 K, where NbTi cables are shaped as half-wavelength waveguide resonators (*19*). On Alice’s side, we use another hybrid ring to entangle a weak coherent state, which serves as the unknown input state, with the shared TMS state. The outputs of this second hybrid ring are guided into a pair of measurement JPAs that perform a strong phase-sensitive amplification with the same gain *G* but along orthogonal amplification angles. By superimposing the outputs of the measurement JPAs at the third hybrid ring, we produce the feedforward signal and conclude step (ii). To understand the final part of our QT protocol, it is useful to consider a combined action $\hat{H}$ of one of the measurement JPAs with Alice’s hybrid rings and Bob’s directional coupler with a coupling constant β = −15 dB. In the ideal case of zero transmission losses *L* = 0, $\hat{H}$ corresponds to a projective operation when the measurement gain *G* exactly compensates for the finite coupling β and path losses of two hybrid rings (3 dB each). The corresponding optimal gain is *G*_opt_ ≃ −β + 6 dB = 21 dB, in the limit of *S* → ∞. By using the input-output formalism for covariance matrices describing a particular input signal path (depicted by the red dashed line in Fig. 1B), one can show that the combined operator $\hat{H}$ is

$$\hat{H}=\frac{\sqrt{\beta }}{2}\hat{J}\overset{\beta \to 0}{\underset{G\cdot \beta \to 4}{\to }}{\hat{\Pi }}_{p}=(\begin{array}{cc} 0 & 0\\ 0 & 1 \end{array}),\hat{J}=(\begin{array}{cc} 1/\sqrt{G} & 0\\ 0 & \sqrt{G} \end{array})$$ (1)

where ${\hat{\Pi }}_{p}$ corresponds to a projective measurement of the *p* quadrature and $\hat{J}$ describes the phase-sensitive amplification by one of the measurement JPAs. Analogously, the other measurement JPA performs projection onto the *q* quadrature and implements the ${\hat{\Pi }}_{q}$ operation. The resulting feedforward application to Bob’s part of the TMS state implements teleportation of the input state at the output of the directional coupler, concluding step (iii) of the QT protocol (see note S3 for detailed calculations). The demonstrated analog feedforward is fully deterministic and allows us to tune the strength of our measurement operation by changing the gain *G* in situ and observe its impact on the teleportation protocol. Last but not least, our analog feedforward avoids many issues related to digital feedforward approaches, such as detection inefficiencies or unavoidable temporal delays due to signal processing.

From a fundamental point of view, the role of the feedforward signal is to overcome arbitrary losses in the classical communication (feedforward) channel. This task is achieved by converting a part of the quantum state information into a strongly amplified classical signal. Then, the measurement (feedforward) gain is optimized to compensate for the feedforward losses and transfer this classical signal safely to Bob. There, in combination with the entangled resource and appropriate local operation, the feedforward signal results in successful QT. However, it is important to remember that even the optimized measurement gain cannot compensate for all transmission losses, especially not for those occurring during the entanglement distribution stage. The reason for this is that local operations cannot compensate for the loss of bipartite mutual information (i.e., quantum correlations).

### QT measurements

**Fig. 2.**
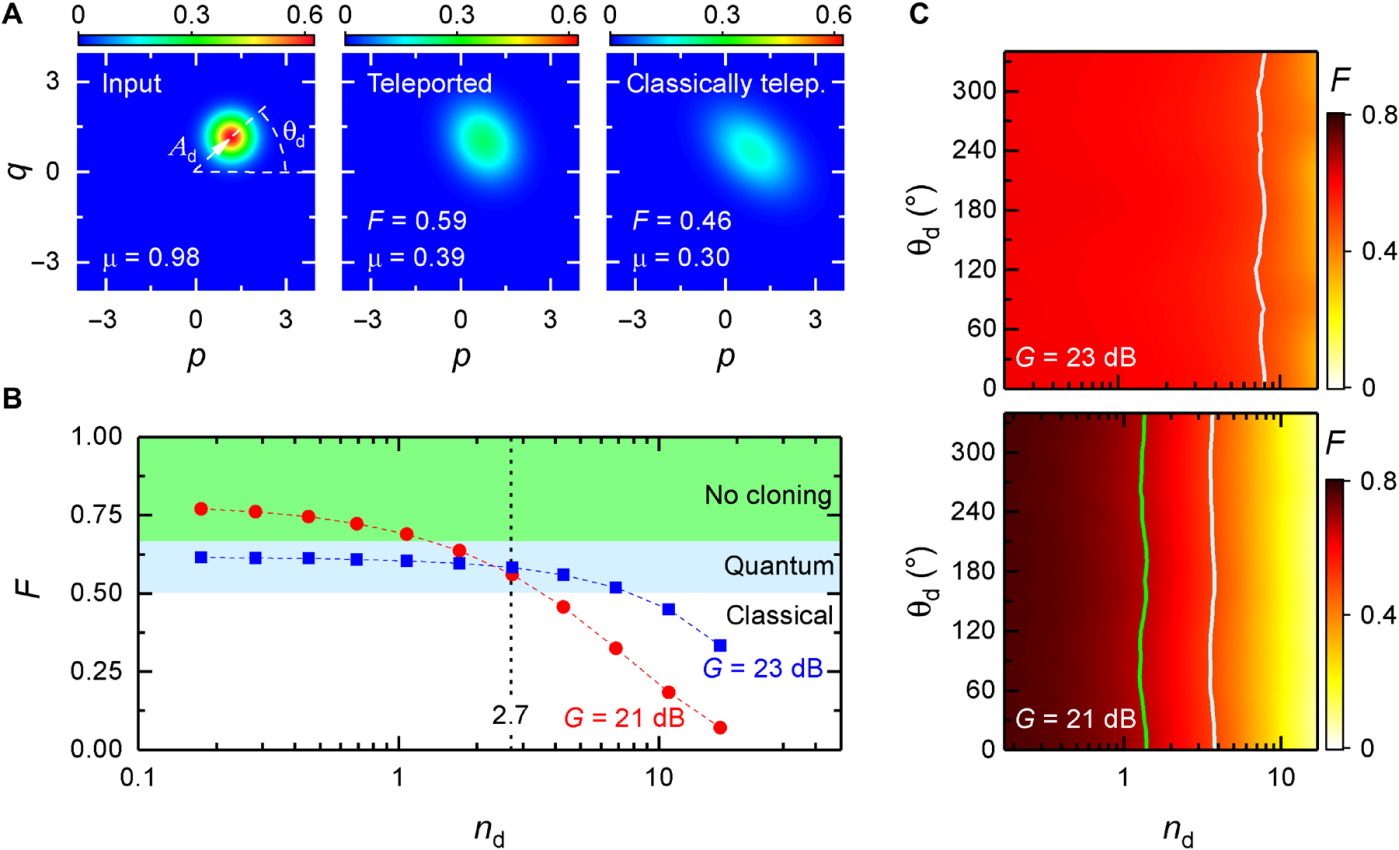
Tomography and fidelity measurements. (**A**) Reconstructed Wigner functions of an input state, teleported state, and classically teleported state for the squeezing level *S* = 4.5 dB, the displacement photon number of the input state *n*_d_ = 2.7, and the measurement gain *G* = 23 dB. Inset values represent the QT fidelity *F* and purity μ. (**B**) Fidelity *F* as a function of *n*_d_ for two characteristic values of *G*. Black dashed line marks the operating point illustrated in (A). The statistical error is smaller than the symbol size. (**C**) Fidelity *F* as a function of *n*_d_ and displacement angle θ_d_ for two characteristic values of *G*. Light blue and green lines mark the classical and no-cloning limits, respectively.

Figure 2 shows experimental results of the microwave QT protocol. They include Wigner state tomography over a broad range of input coherent states, corresponding quantum-teleported output states, and classically teleported output states (transferred using the same protocol but in the absence of entanglement resource). Here, the carrier frequency of the input, TMS, and teleported states is *f* = 5.435 GHz. Experimental bandwidths of the entanglement and measurement JPAs are 11 and 4 MHz, respectively. However, the actual measurement bandwidth Δ*f* = 400 kHz is limited by a digital finite-impulse response filter in the field programmable gate array (FPGA)-based receiver used for state reconstruction. A particular dataset in Fig. 2A demonstrates experimental tomography results with the QT fidelity *F* = 0.596 ± 0.004 larger than the classical threshold *F*_ct_ = 1/2. At the same time, classical teleportation yields *F* = 0.46 < *F*_ct_. Here, we define classical teleportation as the identical QT protocol but with no entangled resource available to the communicating parties (Alice and Bob). It is implemented by switching off the pump tones of entanglement JPAs, while the rest of the experimental protocol remains unaltered.

Strictly speaking, teleportation of a particular coherent state is not sufficient for general purposes of quantum communication. Typically, one needs to demonstrate the successful teleportation of a set of quantum states that could form a communication alphabet (i.e., a codebook). It is important to mention that orthogonality of quantum states forming the communication codebook is not required. Moreover, such orthogonality can be even detrimental for many quantum communication protocols because it compromises their unconditional security. The latter relies on the no-cloning theorem in combination with an eavesdropper inability to distinguish certain quantum states with a single measurement. In other words, if the codebook states are strictly orthogonal, then the respective no-cloning fidelity threshold becomes *F*_nc_ = 1, as the corresponding states are perfectly distinguishable with a single optimal measurement (*20*). In our case, we form the communication codebook with a set of weak coherent states by varying both their phase θ_d_ and amplitude $A_{d}∼\sqrt{n_{d}}$, where *n*_d_ is the displacement photon number. We observe an approximately constant fidelity *F* ≃ 0.55 of the teleported coherent states up to *n*_d_ ≃ 2 photons over the whole phase range for *G* = 23 dB, as shown in Fig. 2C. Moreover, for the measurement gain *G* = 21 dB, we are also able to violate the no-cloning bound *F*_nc_ = 2/3 for *n*_d_ ≤ 1.1 (see Fig. 2B), which is key to the unconditional security of quantum communication (*21*). For *n*_d_ > 1.1, we observe a smooth degradation of teleportation fidelities, which we attribute to compression effects in the measurement JPAs. These compression effects usually arise because of higher-order nonlinearities in strongly driven JPAs (*22*) and mainly affect the measurement JPAs in our experiment. In principle, to make our QT protocol completely secure against potential eavesdropping of the feedforward signal, we would have to extend the power range of teleported states to *n*_d_ ≫ 1 while preserving *F* > *F*_nc_ (*23*). This goal can be achieved in the future by improving the 1-dB compression point of our JPAs, *P*_1−dB_ ≃ −130 dBm, to higher values (*24*). A weak dependence of the QT fidelity *F* on the coherent state phase θ_d_, observable for *n*_d_ ≫ 1 in Fig. 2C, arises from an interplay between the compression effects and nonperfect orthogonality of phase-sensitive amplification in the measurement JPAs.

**Fig. 3.**
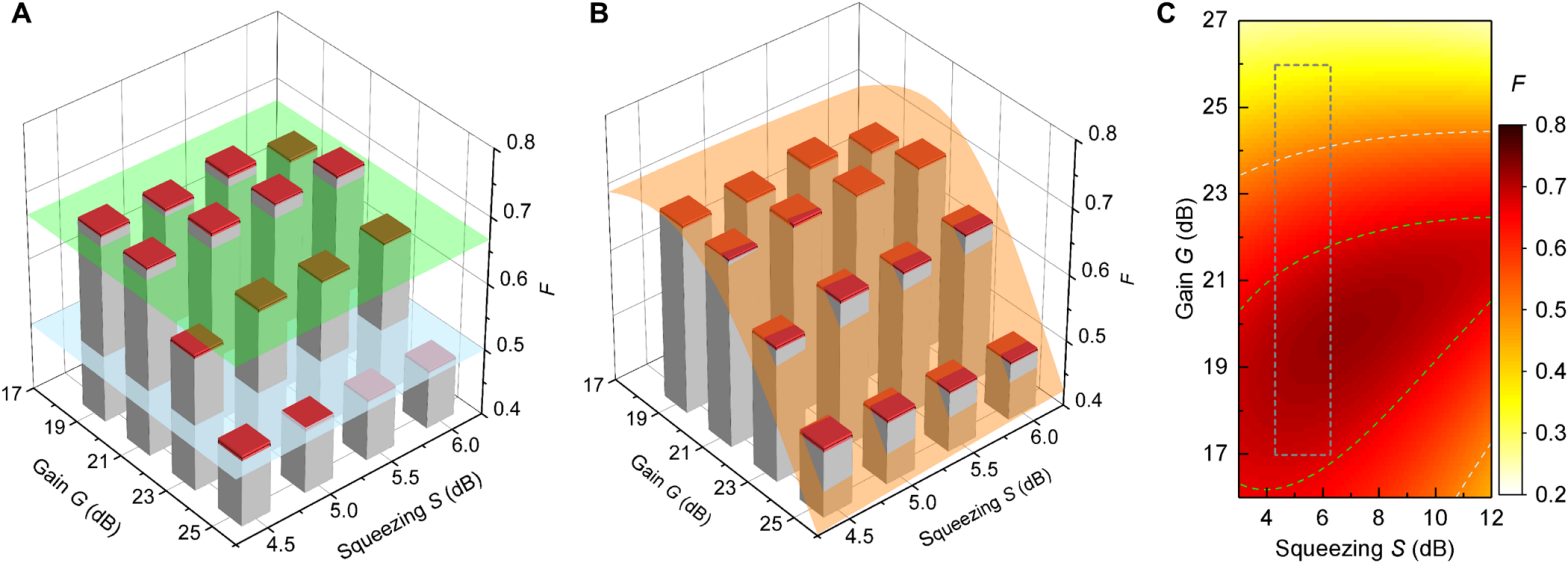
Fidelity thresholds and theory model. (**A**) Experimental QT fidelities *F* as a function of the measurement gain *G* and squeezing *S* for *n*_d_ = 1.1 photons. Red bars denote SD of the experimental data. Light blue plane corresponds to the fidelity threshold *F* = 0.5 between quantum and classical regimes, while green plane denotes the no-cloning limit *F*_nc_ = 2/3. The experimental data violate the no-cloning limit for *G* = 21 dB in the whole range of squeezing levels. (**B**) Same data with the fitted theory model (orange plane). (**C**) Extended view over the expected QT performance for the same model, where dark gray dashed box outlines the area presented in (B). This theory plot demonstrates that further improvement of teleportation fidelities requires an increase of both the measurement gain *G* and squeezing level *S*.

Last, we investigate the influence of both the measurement JPA gain *G* and entanglement strength expressed via the squeezing level *S* on the QT performance. This task is straightforwardly achieved in our experimental setup by tuning the microwave pump amplitudes of the measurement and entanglement JPAs. Figure 3A shows the experimental fidelity *F* of coherent state teleportation as a function of *G* and *S*. One can immediately notice a fidelity maximum of *F* = 0.689 ± 0.004 at *G* = 21 dB as naively expected from theory. However, one has to keep in mind that *G*_opt_ = 21 dB is only valid in the limit of *S* → ∞ and *L* = 0. In reality, our squeezing levels are not infinite. In addition, we estimate microwave losses *L* ≃ 2 dB due to reflections and dissipation in nonsuperconducting parts. To describe these nonidealities, we develop a theory model on the basis of the input-output formalism and fit it to the experimental data. This fit has three fitting parameters: temperature *T* of the electromagnetic environment and noise parameters χ_1_ and χ_2_ that describe the JPA gain dependence of the noise photon number *n* = χ_1_(*G* − 1)^χ_2_^. Here, *n* is referred to the JPA input and *G* corresponds to the JPA degenerate gain (*25*). All other model parameters, such as various losses, are determined from independent measurements and can be found in note S2. We find a good qualitative agreement between theory and experiment, as it can be seen in Fig. 3B. Our model also demonstrates that the optimal measurement gain is weakly dependent on *S* and becomes $G_{\text{opt}}^{\prime }=23\;\text{dB}\;$ for *S* → ∞. This can be intuitively understood by considering the fact that an asymptotic optimal gain also needs to compensate for finite transmission losses $G_{\text{opt}}^{\prime }=G_{\text{opt}}+L$, while *G* = 21 dB appears to be only a local optimum for the finite squeezing levels accessible in our experiments. This optimum also becomes more pronounced if one considers future possible improvements of the current protocol with reduced transmission losses or reduced JPA noise (see note S5). This observation is also in agreement with the fact that the optimal gain increases with the increasing entanglement strength (*26*).

## DISCUSSION

### Teleportation bit rate

To quantify the amount of information, which can be sent via QT of coherent states, we use the effective number of classical bits encoded in one of the teleported state parameters. Here, we focus on the coherent state phase θ_d_, because it is most suitable for communication of classical information for a fixed *n*_d_. We estimate the corresponding bit rate *N* in our QT protocol by using the well-known Shannon-Hartley equation for the channel capacity, $N=\Delta f{\text{log}}_{2}(1+\text{SNR})$. In our case, the signal-to-noise ratio (SNR) can be expressed as $\text{SNR}=\Delta \theta_{d}^{2}/\sigma^{2}$, where Δθ_d_ is the available range of coherent phases and σ is the SD of θ_d_. To obtain a lower bound for σ, we use the quantum Cramér-Rao bound (*27*), which relates the quantum Fisher information ℱ_Q_ to a lower bound of the estimation error of θ_d_ as σ^2^ ≥ 1/ℱ_Q_. This bound can be saturated by a suitable measurement choice. The quantum Fisher information ℱ_Q_ for phase estimation of a displaced Gaussian state can be straightforwardly obtained from the tomography parameteres of teleported states, as described in (*28*). For our QT settings corresponding to the working point with *G* = 21 dB, *S* = 6 dB, and *n*_d_ = 1.1, we obtain ℱ_Q_ ≃ 0.85. From Fig. 2, we see that Δθ_d_ = 2π, which results into the upper bound for the achievable bit rate *N* = 1.95 Mbits/s. In practice, to achieve this upper bound, one must implement an appropriate optimal measurement that allows to saturate the particular quantum Fisher information. For phase estimation of coherent states, this optimal measurement consists of (i) rough estimation of an unknown phase θ, followed by (ii) optimizing quadrature measurements along the direction θ + π/2 in the phase space (*20*). Because the current experiment does not aim at implementing this optimal measurement, one can only give an order of magnitude estimation of an actual bit rate *N*_a_. This estimation can be achieved by exploiting a measurement quantum efficiency η ≃ 4 %, which is extracted from the photon number calibration measurements. Thus, the actual bit rate encoded in the teleported, phase-modulated, noisy coherent states that are probed with the noisy quadrature measurements (under otherwise ideal conditions) can be estimated as *N*_a_ ≃ *N* · η = 78 kbits/s.

Last, it is important to mention a connection between the bit rate *N* and an unconditionally secure bit rate *N*_s_. The latter, in the current experiment, can become positive, *N*_s_ > 0, only in the situation when the teleportation fidelity exceeds the no-cloning bound for a particular codebook, *F* > *F*_nc_. In the case of ideal teleportation, *F* → 1, these two bit rates converge, *N*_s_ → *N*. In a more realistic scenario of finite fidelites exceeding the no-cloning bound, 1 > *F* > *F*_nc_, the task of estimating the realistic secret key rate requires a careful analysis with extra assumptions about possible attacks from a third party (eavesdropper) (*29*).

In conclusion, we have successfully implemented the QT protocol with propagating microwaves over a distance of 0.42 m in the cryogenic environment. Our teleportation protocol allows us to violate the no-cloning limit over a wide range of input state parameters, corresponding to teleportation fidelities of *F* ≥ 0.69 for coherent states with the displacement photon number of *n*_d_ ≤ 1.1 and the measurement gain *G* = 21 dB. Our experimental techniques rely exclusively on conventional aluminum-niobium superconducting parametric devices for generation and control of quantum microwave signals, which makes them fully compatible with other quantum superconducting circuits in terms of frequencies and fabrication technology, such as microwave quantum memory cells (*30*, *31*) or superconducting quantum processors (*32*). This natural technology match also avoids massive (∼10^−5^) conversion losses of state-of-the-art transducers between optical and microwave frequencies at the single photon level (*33*). On the basis of the experimental parameters, our theory model predicts that the current QT implementation can tolerate up to ≃1 dB of extra losses in both entanglementdistribution channels (see Fig. 1B) before the QT fidelity drops below the no-cloning threshold *F*_nc_. These additional losses translate into an extra communication distance of approximately ≃1 km. Correspondingly long superconducting microwave cables can be already manufactured by connecting standard, commercially available, 1- to 2-m pieces of superconducting NbTi coaxial cables with point-welded NbTi superconducting joints. Our preliminary tests have indicated sufficiently small microwave propagation losses even in the presence of these joints. Therefore, we envision a near-term possibility for fabrication of sufficiently long, up to 1 km, superconducting cables. Moreover, a technological backbone for such superconducting quantum networks is already available in the form of microwave cryogenic links. These cryolinks have several promising advantages over optical links: (i) very high quantum efficiencies for single-photon transmission due to the absence of frequency transduction and (ii) comparatively straightforward technical implementation (*34*). The demonstrated microwave QT results, in combination with the aforementioned technological advances, bring quantum local area networks between superconducting quantum computers withing reach. Furthermore, our experiments pave the road toward distributed superconducting quantum supercomputers and allow one to exploit advantages of secure quantum communication in the convenient microwave regime.

## MATERIALS AND METHODS

The task of the entanglement JPAs is to perform a local squeezing operation $\hat{S}(\xi )|0\rangle$, which can be described by a single-mode squeezing operator $\hat{S}(\xi )=\text{exp}\;(\frac{1}{2}\xi^{*}{\hat{a}}^{2}-\frac{1}{2}\xi {\left({\hat{a}}^{†}\right)}^{2})$, where ${\hat{a}}^{†}=\hat{q}-i\hat{p}$ and $\hat{a}=\hat{q}+i\hat{p}$ are the creation and annihilation operators with $[\hat{a},{\hat{a}}^{†}]=1$ of the *f* mode with quadrature operators $\hat{q}$ and $\hat{p}$ and ξ = *re*^*i*ϕ^ is the complex squeezing amplitude. Here, the phase ϕ = −2γ determines the squeezing angle γ between the antisqueezed quadrature and the *p* axis in the phase space, while the squeezing factor *r* parameterizes the amount of squeezing. We define the squeezing level in decibels as $S=-10\;{\text{log}}_{10}(\sigma_{s}^{2}/0.25)$, where $\sigma_{s}^{2}$ is the variance of the squeezed quadrature and the vacuum variance is 0.25. Positive values of *S* indicate squeezing below the vacuum level. The antisqueezing level is defined as $A=10\;{\text{log}}_{10}(\sigma_{a}^{2}/0.25)$, where $\sigma_{a}^{2}$ is the variance of the antisqueezed quadrature. We generate symmetric TMS states at the output of the hybrid ring by pumping entanglement JPAs 1 and 2 with strong quasi-continuous microwave drives so that they produce steady-state squeezed vacuum states with the same squeezing level but orthogonal squeezing angles, γ_2_ = γ_1_ + π/2. These angles are stabilized by controlling the respective pump phases using a phase-locked loop (*17*, *35*). A TMS state can be described by the two-mode squeezing operator ${\hat{S}}_{T}=\text{exp}\;(\xi_{T}^{*}{\hat{a}}_{1}{\hat{a}}_{2}-\xi_{T}{\hat{a}}_{1}^{†}{\hat{a}}_{2}^{†})$, where ${\hat{a}}_{i}$ is the annihilation operator of the *i*th electromagnetic mode and ξ_T_ = *r*_T_*e*^*i*φ^. Here, the amount of two-mode squeezing is given by $S_{T}=-10\;{\text{log}}_{10}(\text{exp}\;(-2r_{T}))$ and the phase φ determines which quadratures of the two modes are correlated. The difference between the genuine two-mode squeezing level *S*_T_ (at the hybrid ring outputs) and local squeezing level *S* (at the hybrid ring inputs) is fully defined by the hybrid ring insertion losses *L*_HR_ = 0.4 dB and environmental noise photon number *n*_env_ ≃ 0.025. In our case, these effects amount to a small difference of roughly 10%. Thus, we can use *S* as a good direct quantifier for the amount of two-mode squeezing in the propagating microwave signals.

An important technical aspect of the microwave TMS state generation is balancing–symmetrizing of local and nonlocal state variances (*17*). In our experiment, balancing is achieved by fine tuning of both the phase and amplitude of independent pump tones of the of entanglement JPAs. The latter step is also crucial to compensate for a pump cross-talk, which may alter the squeezing levels and angles of the interconnected JPAs. This happens due to the fact that strong pump tones (at the frequencies of ∼11 GHz) leak through the input JPA circulators, which are specified only for the frequency range of 4 to 8 GHz, and affect other JPAs. Typically, we run a complicated pump pulse sequence to calibrate this cross-talk and minimize it. A more universal solution would be to use broadband cryogenic circulators (with the frequency range of 4 to 12 GHz) to directly suppress this cross-talk.

All four JPAs used in the current experiments are nominally identical. The spread of experimentally extracted JPA parameters, such as internal and external quality factors or bare resonator frequencies, is on the order of 15%. Internal quality factors of these JPAs, *Q*_int_ ≃ 5 · 10^4^, play an important role in achieving reasonable squeezing levels, *S* ∼ 6 dB, with low added noise photon numbers, *n*_n_ ≲ 0.03. This combination is the key to obtain high teleportation fidelities. In principle, generation of these microwave TMS states can be also achieved by using other superconducting quantum devices, such as Josephson parametric converters (JPCs) (*36*, *37*), which may help to reduce total transmission losses by effectively removing lossy hybrid rings. However, an experimental implementation of frequency-degenerated JPCs, as required in our teleportation protocol, is missing at this moment.

To reconstruct various quantum states in the experiment, we use a well-tested reference state tomography on the basis of statistical moments of the detected field quadratures of Gaussian states (*16*, *38*). We verify the Gaussian character of experimentally detected states by checking their cumulants (see note S4). All corresponding error bars provided in the current manuscript are based on the statistical uncertainty in the form of SD of the measurement statistics. The accuracy of the system calibration and tomography measurements directly depends on the accuracy of the photon number calibration, which is based on Planck spectroscopy (*39*). The accuracy of this calibration in our experiments (i.e., the accuracy of the voltage-to-photon-number conversion coefficient) is typically around 1%. Numerical analysis indicates that this calibration inaccuracy roughly corresponds to systematic errors of the teleportation fidelities on the order of 0.3%.

## Supplementary Materials

### This PDF file includes:

Notes S1 to S5 Figs. S1 to S5 Table S1
