# Supplementary material for: Experimental quantum teleportation of propagating microwaves
Source: Sci Adv. 2021 Dec 22;7(52):eabk0891. doi: 10.1126/sciadv.abk0891 (PMC8694421; doi:10.1126/sciadv.abk0891)
Supplement: Supplementary file 1 — Notes S1 to S5 Figs. S1 to S5 Table S1 [file sciadv.abk0891_sm.pdf]

Science Advances
AAAS

Supplementary Materials for

Experimental quantum teleportation of propagating microwaves

Kirill G. Fedorov*, Michael Renger, Stefan Pogorzalek, Roberto Di Candia, Qiming Chen, Yuki Nojiri, Kunihiro Inomata, Yasunobu Nakamura, Matti Partanen, Achim Marx, Rudolf Gross, Frank Deppe

*Corresponding author. Email: kirill.fedorov@wmi.badw.de

Published 22 December 2021, *Sci. Adv.* **7**, eabk0891 (2021)
DOI: 10.1126/sciadv.abk0891

This PDF file includes:

Notes S1 to S5
Figs. S1 to S5
Table S1

Supplementary materials

Supplementary note 1: Experimental Setup

The experimental room temperature and cryogenic setup is shown in Figure S1. The FPGA and the microwave pump sources for each JPA are pulsed with a data timing generator (DTG). JPA 1 and JPA 2 are both temperature stabilized at $50\,\mathrm{mK}$ in order to ensure a stable JPA operation and produce squeezed states with orthogonal squeezing angles. The two squeezed states are superimposed by a cryogenic hybrid ring (50:50 beam splitter) in order to produce path-entangled two-mode squeezed (TMS) states at the outputs of the hybrid ring. By operating JPA 1 and JPA 2 at the same squeezing level, we are able to produce symmetric TMS states with local statistics of a thermal state in each single path. One output path of the beam splitter is connected to a directional coupler while the other one is connected to a cryogenic switch allowing us to either directly detect the signal for characterization purposes or perform quantum teleportation by sending it to another beam splitter. Here, one part of the TMS state is superimposed with the input signal (to-be-teleported signal) and each of the outputs is sent to measurement JPAs (JPA 3 and JPA 4) for strong phase-sensitive amplification with orthogonal amplification angles. Afterwards, another cryogenic switch allows for choosing between a characterization configuration or sending both amplified signals to a third beam splitter. One output of the beam splitter is connected to the coupled port of a directional coupler. For all output lines, the first amplification stage of a high-electron-mobility transistor (HEMT) operating at low temperatures is followed by a room temperature rf-amplifier, which is temperature-stabilized by a Peltier cooler. We use a vector network analyzer for a spectroscopic characterization of the JPAs and a heterodyne detection setup for the tomographic measurements.

The heterodyne detection setup and data processing are similar to those described in Refs. (*17, 18*). Here, the signal is roughy filtered around the working frequency and down-converted to

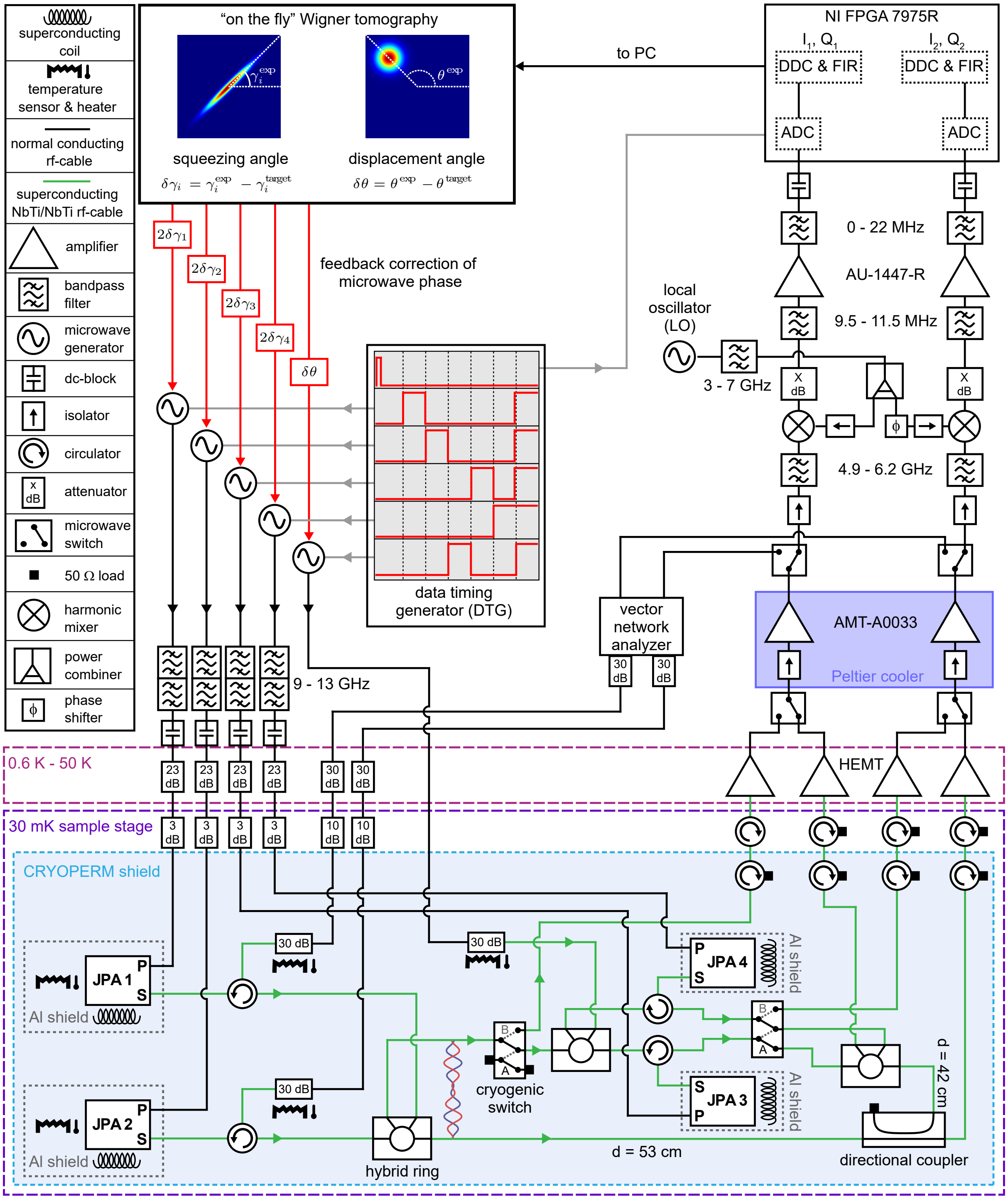

Figure S1: **Experimental scheme of microwave quantum teleportation protocol with propagating states.** The two cryogenic switches allow for quantum teleportation (switch position A) or characterization measurements (switch position B). The intertwined lines between the outputs of the hybrid ring symbolize the entanglement.

$11\,\mathrm{MHz}$ by image rejection mixers. The signal is then digitized by a National Instruments NI-5782 transceiver module and processed with a National Instruments PXIe-7975R FPGA module in real time. The digital data processing consists of digital down-conversion (DDC), finite-impulse response (FIR) filtering with a full bandwidth of $400\,\mathrm{kHz}$, and calculation as well as averaging of all quadrature moments $\langle I_1^n I_2^m Q_1^k Q_2^l \rangle$ with $n+m+k+l \leq 4$ for $n,m,k,l \in \mathbb{N}$. During each measurement cycle, the moments of JPAs 1-4 are used to calculate the squeezing angles γ_i^{exp} for each JPA "on the fly" in order to obtain the angle correction $\delta\gamma_i = \gamma_i^{\mathrm{ext}} - \gamma_i^{\mathrm{target}}$ which is used to adjust the phase of the microwave pump tone by $2\delta\gamma_i$. Similarly, the phase of the microwave signal tone is adjusted by $\delta\theta = \theta^{\mathrm{ext}} - \theta^{\mathrm{target}}$ where θ is the displacement angle of the input coherent state for quantum teleportation. During data analysis on the PC, the recorded traces are separated into different parts according to the DTG timings as indicated in Figure S1. The data within a single averaging cycle consists of 7.7×10^8 raw data points per part of the trace and is used to perform a reference state reconstruction for each pulse in order to obtain the signal moments $\langle (\hat{a}^\dagger)^n \hat{a}^m \rangle$ with $n+m \leq 4$. Finally, this averaging cycle is repeated 10 times. The vector network analyzer, DTG, FPGA card and local oscillator are synchronized to a $10\,\mathrm{MHz}$ rubidium frequency standard. The pump microwave sources are daisy chained to the local oscillator with a $1\,\mathrm{GHz}$ reference signal.

Supplementary note 2: Theory Model

The quantum teleportation protocol is theoretically modelled in an iterative way, as illustrated in Figure S2. The scheme consists of 3 signal paths where path 1 and path 2 form the input for the TMS generation and the input signal is applied at path 3. The weak thermal environment in each path is modelled by the bosonic operator $\hat{f}$ with the average noise photon number

$$\langle \hat{f}^\dagger \hat{f} \rangle = n_{\mathrm{th}} = \frac{1}{e^{\frac{\hbar\omega}{k_{\mathrm{B}}T}} - 1}. \tag{S1}$$

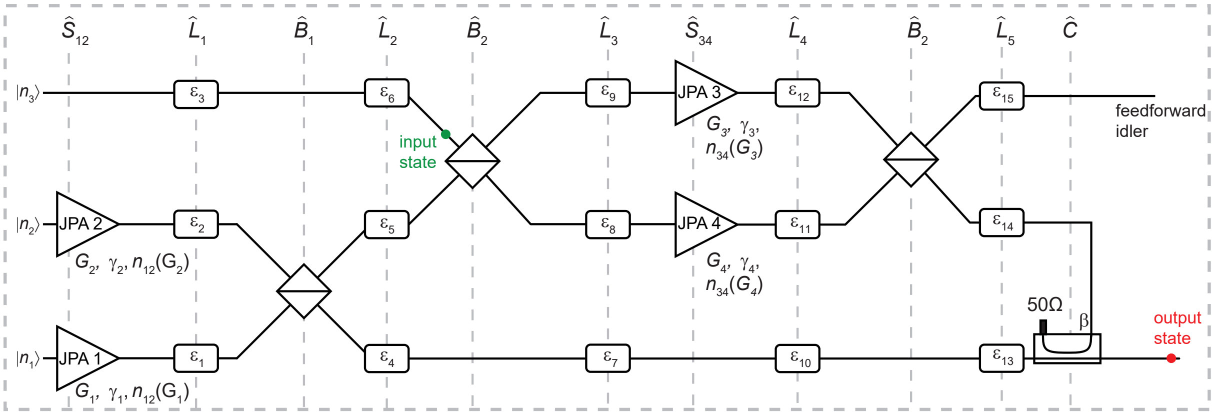

Figure S2: **Scheme for the simulation of the quantum teleportation protocol.** Each iteration step consists either of a unitary operation such as squeezing ($\hat{S}_{12}$, $\hat{S}_{34}$) and beam splitter operations ($\hat{B}_1$, $\hat{B}_2, \hat{C}$), or of a non-unitary operation such as path losses ($\hat{L}_1, \hat{L}_2, \hat{L}_3, \hat{L}_4, \hat{L}_5$) and added noise. The squeezing parameter of JPA 1 (JPA 2) is denoted by r_1 (r_2) and γ_1 (γ_2) is the squeezing angle in radians. The degenerate gain of JPA 3 (JPA 4) is described by G_3 (G_4) and γ_3 (γ_4) is the respective squeezing angle in radians. The input state is reconstructed at the position indicated by green dot. Red dot denotes the reconstruction point for the teleported output state.

Weak thermal states form inputs for squeezed state generation with JPA 1 and JPA 2 and subsequent path-entanglement at the outputs of the hybrid ring. As a result, the input noise photon numbers n_1 and n_2 consist of the environmental thermal noise and the noise added by the JPAs, which monotonically increases with increasing degenerate gain G_i, $i \in \{1, 2\}$. Based on the existing experimental evidence and related phenomenological theory (*25*), we assume that this gain dependence obeys a power law

$$n_1 = n_{\text{th}} + \chi_1(G_1 - 1)^{\chi_2}, \qquad n_2 = n_{\text{th}} + \chi_1(G_2 - 1)^{\chi_2}. \tag{S2}$$

The initial thermal coherent state $\hat{a}$ with displacement α in path 3 is modelled by applying the displacement operator $\hat{D}(\alpha)$ to the operator $\hat{f}_3$ which describes the initial bosonic noise

$$\hat{a} = \hat{D}(\alpha)^\dagger \hat{f}_3 \hat{D}(\alpha) = \hat{f}_3 + \alpha. \tag{S3}$$

The number of noise photons n_3 in the coherent input is quantified by the purity $\mu = 1/(1 + 2n_3)$. As a result, we formally write the input state as $|n_1; n_2; \alpha, \mu\rangle \equiv |n_1\rangle \otimes |n_2\rangle \otimes |\alpha, \mu\rangle$. In

each iteration step, we apply either a unitary squeezing/beam splitter operation or a non-unitary operation to model losses and noise. In the first step, a thermal squeezed state is created by JPA 1 and JPA 2. This operation is modelled with the squeezing operator $\hat{S}_{12}$, acting on path 1 and path 2

$$\hat{S}_{12}^\dagger \begin{pmatrix} \hat{a}_1 \\ \hat{a}_2 \\ \hat{a}_3 \end{pmatrix} \hat{S}_{12} = \begin{pmatrix} \hat{a}_1 \cosh r_1 - \hat{a}_1^\dagger e^{-2i\gamma_1} \sinh r_1 \\ \hat{a}_2 \cosh r_2 - \hat{a}_2^\dagger e^{-2i\gamma_2} \sinh r_2 \\ \hat{a}_3 \end{pmatrix} , \tag{S4}$$

where $\hat{a}_j$ is the signal operator in path j. In the next step, path 1 and path 2 are entangled with a hybrid ring, which we model as a 50:50 beam splitter by

$$\hat{B}_1^\dagger \begin{pmatrix} \hat{a}_1 \\ \hat{a}_2 \\ \hat{a}_3 \end{pmatrix} \hat{B}_1 = \frac{1}{\sqrt{2}} \begin{pmatrix} \hat{a}_1 + \hat{a}_2 \\ -\hat{a}_1 + \hat{a}_2 \\ \sqrt{2}\, \hat{a}_3 \end{pmatrix} . \tag{S5}$$

The losses are modelled with a beam splitter model according to

$$\hat{L}_j^\dagger \begin{pmatrix} \hat{a}_1 \\ \hat{a}_2 \\ \hat{a}_3 \end{pmatrix} \hat{L}_j = \begin{pmatrix} \sqrt{1-\varepsilon_{3(j-1)+1}}\, \hat{a}_1 + \sqrt{\varepsilon_{3(j-1)+1}}\, \hat{v}_{3(j-1)+1} \\ \sqrt{1-\varepsilon_{3(j-1)+2}}\, \hat{a}_2 + \sqrt{\varepsilon_{3(j-1)+2}}\, \hat{v}_{3(j-1)+2} \\ \sqrt{1-\varepsilon_{3(j-1)+3}}\, \hat{a}_3 + \sqrt{\varepsilon_{3(j-1)+3}}\, \hat{v}_{3(j-1)+3} \end{pmatrix} \tag{S6}$$

with $j \in \{1, 2, 3, 4, 5\}$, cf. Figure S2. The bosonic bath modes $\hat{v}_{3(j-1)+1}$ model the thermal environment. The power losses are denoted by $\varepsilon_{3(j-1)+3}$. The 50:50 beam splitter which entangles path 2 and path 3 is modelled as

$$\hat{B}_2^\dagger \begin{pmatrix} \hat{a}_1 \\ \hat{a}_2 \\ \hat{a}_3 \end{pmatrix} \hat{B}_2 = \frac{1}{\sqrt{2}} \begin{pmatrix} \sqrt{2}\, \hat{a}_1 \\ \hat{a}_2 + \hat{a}_3 \\ -\hat{a}_2 + \hat{a}_3 \end{pmatrix} . \tag{S7}$$

In the next step, JPAs 3 (JPA 4) acts as a degenerate parametric amplifier to realize the Bell measurement by amplifying orthogonal signal quadratures with respective degenerate gain G_3 (G_4). This amplification process is modelled with a squeezing operator, acting on path 2 and path 3, according to

$$\hat{S}_{34}^\dagger \begin{pmatrix} \hat{a}_1 \\ \hat{a}_2 \\ \hat{a}_3 \end{pmatrix} \hat{S}_{34} = \begin{pmatrix} \hat{a}_1 \\ (\hat{a}_2 + \zeta_3) \cosh r_3 - (\hat{a}_2^\dagger + \zeta_3^*) e^{-2i\gamma_3} \sinh r_3 \\ (\hat{a}_3 + \zeta_4) \cosh r_4 - (\hat{a}_3^\dagger + \zeta_4^*) e^{-2i\gamma_4} \sinh r_4 \end{pmatrix} , \tag{S8}$$

where the squeezing parameters r_3 (r_4) are related to the degenerate gain via $G_3 = e^{2r_3}$ ($G_4 = e^{2r_4}$) and the squeezing angle of JPA 3 (JPA 4) is denoted by γ_3 (γ_4). In the following, we assume equal degenerate gain for both JPAs, $G_3 = G_4 = G$. The noise added by JPA 3 (JPA 4) is modelled with a random classical variable ζ_3 (ζ_4). We assume that JPA 3 and JPA 4 have equal noise properties, $\zeta_3 = \zeta_4 = \zeta$ and assume ζ to obey a centralized Gaussian distribution with $\langle \zeta\zeta^* \rangle = n_{34}(G)$ and $\langle \mathrm{Re}(\zeta^2) \rangle = \langle \mathrm{Im}(\zeta^2) \rangle = n_{34}(G)/2$. Similar as in Eq. S2, we assume that the JPA noise depends on the gain G as

$$n_{34}(G) = \chi_1 (G-1)^{\chi_2}, \tag{S9}$$

where χ_1 and χ_2 are phenomenological constants characterizing noise properties of the JPAs. The displacement operation for Bob's quantum state in path 1 is realized by a directional coupler, acting as an asymmetric beam splitter with a reflectivity β

$$\hat{C}^\dagger \begin{pmatrix} \hat{a}_1 \\ \hat{a}_2 \\ \hat{a}_3 \end{pmatrix} \hat{C} = \begin{pmatrix} \sqrt{1-\beta}\,\hat{a}_1 + \sqrt{\beta}\,\hat{a}_2 \\ -\sqrt{\beta}\,\hat{a}_1 + \sqrt{1-\beta}\,\hat{a}_2 \\ \hat{a}_3 \end{pmatrix}. \tag{S10}$$

In the ideal case, as it will be shown later, β needs to fulfill the analog teleportation condition $\beta = 4/[g(1-\varepsilon)]$, where ε models the path losses after JPA 3 and JPA 4. Finally, the teleportation protocol can be expressed by the operator

$$\hat{T} = \hat{C}\hat{L}_5\hat{B}_2\hat{L}_4\hat{S}_{34}\hat{L}_3\hat{B}_2\hat{L}_2\hat{B}_1\hat{L}_1\hat{S}_{12}, \tag{S11}$$

and the final state $|\Psi\rangle$ is given by

$$|\Psi\rangle = \hat{T}|n_1; n_2; \alpha, n_3\rangle. \tag{S12}$$

The moments of the output signal $\hat{b}$ can then be calculated by

$$\begin{pmatrix} \langle (\hat{b}^\dagger)^n \hat{b}^m \rangle_1 \\ \langle (\hat{b}^\dagger)^n \hat{b}^m \rangle_2 \\ \langle (\hat{b}^\dagger)^n \hat{b}^m \rangle_3 \end{pmatrix} = \langle \Psi | \begin{pmatrix} \langle (\hat{a}^\dagger)^n \hat{a}^m \rangle_1 \\ \langle (\hat{a}^\dagger)^n \hat{a}^m \rangle_2 \\ \langle (\hat{a}^\dagger)^n \hat{a}^m \rangle_3 \end{pmatrix} |\Psi\rangle. \tag{S13}$$

We assume that all quantum states are Gaussian, which implies that only moments up to second order are required for full state tomography. As a result, it is sufficient to analyze the effect of the teleportation protocol on the respective displacement vector d and covariance matrix V. We realize this by rewriting Eq.S13 specifically for the first- and second-order quadrature moments. For the initial displacement vector d_0, we have

$$d_0 = (0,\ 0,\ 0,\ 0,\ \sqrt{n_\mathrm{d}}\,\cos\varphi_\mathrm{d},\ \sqrt{n_\mathrm{d}}\,\sin\varphi_\mathrm{d})^\mathrm{T} \tag{S14}$$

where n_d is the number of displacement photons and φ_d describes the displacement angle. In the following, I_2 denotes the 2×2 identity matrix and 0_2 is the 2×2 zero matrix. For the initial covariance matrix, we write

$$V_0 = \frac{1}{4}\begin{pmatrix} (1+2n_1)I_2 & 0_2 & 0_2 \\ 0_2 & (1+2n_2)I_2 & 0_2 \\ 0_2 & 0_2 & (1+2n_3)I_2 \end{pmatrix}. \tag{S15}$$

The squeeze operation for JPA 1 and JPA 2 is modelled by

$$S_{12} = \underbrace{\begin{pmatrix} e^{-r_1} & 0 & 0 & 0 & 0 & 0 \\ 0 & e^{r_1} & 0 & 0 & 0 & 0 \\ 0 & 0 & e^{-r_2} & 0 & 0 & 0 \\ 0 & 0 & 0 & e^{r_2} & 0 & 0 \\ 0 & 0 & 0 & 0 & 1 & 0 \\ 0 & 0 & 0 & 0 & 0 & 1 \end{pmatrix}}_{\text{Squeezing with } r_1, r_2} \cdot \underbrace{\begin{pmatrix} \cos 2\gamma_1 & \sin 2\gamma_1 & 0 & 0 & 0 & 0 \\ -\sin 2\gamma_1 & \cos 2\gamma_1 & 0 & 0 & 0 & 0 \\ 0 & 0 & \cos 2\gamma_2 & \sin 2\gamma_2 & 0 & 0 \\ 0 & 0 & -\sin 2\gamma_2 & \cos 2\gamma_2 & 0 & 0 \\ 0 & 0 & 0 & 0 & 1 & 0 \\ 0 & 0 & 0 & 0 & 0 & 1 \end{pmatrix}}_{\text{Rotation by } 2\gamma_1, 2\gamma_2}. \tag{S16}$$

The beam splitter operations can be expressed as

$$B_2 = \frac{1}{\sqrt{2}}\begin{pmatrix} \sqrt{2}I_2 & 0_2 & 0_2 \\ 0_2 & I_2 & I_2 \\ 0_2 & -I_2 & I_2 \end{pmatrix}, \qquad B_1 = \frac{1}{\sqrt{2}}\begin{pmatrix} I_2 & I_2 & 0_2 \\ -I_2 & I_2 & 0_2 \\ 0_2 & 0_2 & \sqrt{2}\,I_2 \end{pmatrix}. \tag{S17}$$

To model the phase-sensitive amplification of JPA 3 and JPA 4, we define the matrices

$$S_3 = \underbrace{\begin{pmatrix} 1 & 0 & 0 & 0 & 0 & 0 \\ 0 & 1 & 0 & 0 & 0 & 0 \\ 0 & 0 & 1/\sqrt{G_3} & 0 & 0 & 0 \\ 0 & 0 & 0 & \sqrt{G_3} & 0 & 0 \\ 0 & 0 & 0 & 0 & 1 & 0 \\ 0 & 0 & 0 & 0 & 0 & 1 \end{pmatrix}}_{\text{Amplification with degenerate gain } G_3}, \qquad R_3 = \underbrace{\begin{pmatrix} 1 & 0 & 0 & 0 & 0 & 0 \\ 0 & 1 & 0 & 0 & 0 & 0 \\ 0 & 0 & \cos 2\gamma_3 & \sin 2\gamma_3 & 0 & 0 \\ 0 & 0 & -\sin 2\gamma_3 & \cos 2\gamma_3 & 0 & 0 \\ 0 & 0 & 0 & 0 & 1 & 0 \\ 0 & 0 & 0 & 0 & 0 & 1 \end{pmatrix}}_{\text{Rotation by } 2\gamma_3}, \tag{S18}$$

and

$$S_4 = \underbrace{\begin{pmatrix} 1 & 0 & 0 & 0 & 0 & 0 \\ 0 & 1 & 0 & 0 & 0 & 0 \\ 0 & 0 & 1 & 0 & 0 & 0 \\ 0 & 0 & 0 & 1 & 0 & 0 \\ 0 & 0 & 0 & 0 & 1/\sqrt{G_4} & 0 \\ 0 & 0 & 0 & 0 & 0 & \sqrt{G_4} \end{pmatrix}}_{\text{Amplification with degenerate gain } G_4}, \qquad R_4 = \underbrace{\begin{pmatrix} 1 & 0 & 0 & 0 & 0 & 0 \\ 0 & 1 & 0 & 0 & 0 & 0 \\ 0 & 0 & 1 & 0 & 0 & 0 \\ 0 & 0 & 0 & 1 & 0 & 0 \\ 0 & 0 & 0 & 0 & \cos 2\gamma_4 & \sin 2\gamma_4 \\ 0 & 0 & 0 & 0 & -\sin 2\gamma_4 & \cos 2\gamma_4 \end{pmatrix}}_{\text{Rotation by } 2\gamma_4}. \tag{S19}$$

The phase-sensitive amplification of JPA 3 and JPA 4 is then described by the matrices

$$J_3 = R_3^\dagger S_3 R_3, \qquad J_4 = R_4^\dagger S_4 R_4. \tag{S20}$$

The noise added by JPA 3 and JPA 4 is described by

$$N_3 = \frac{n_{34}(G_3)}{2} \begin{pmatrix} 0_2 & 0_2 & 0_2 \\ 0_2 & I_2 & 0_2 \\ 0_2 & 0_2 & 0_2 \end{pmatrix}, \qquad N_4 = \frac{n_{34}(G_4)}{2} \begin{pmatrix} 0_2 & 0_2 & 0_2 \\ 0_2 & 0_2 & 0_2 \\ 0_2 & 0_2 & I_2 \end{pmatrix}. \tag{S21}$$

To model the losses, we define the corresponding matrices

$$L_j = \begin{pmatrix} \sqrt{1-\varepsilon_{3(j-1)+1}}\, I_2 & 0_2 & 0_2 \\ 0_2 & \sqrt{1-\varepsilon_{3(j-1)+2}}\, I_2 & 0_2 \\ 0_2 & 0_2 & \sqrt{1-\varepsilon_{3(j-1)+3}}\, I_2 \end{pmatrix} \tag{S22}$$

and

$$A_j = \frac{1}{4}(1+2n_{\mathrm{th}}) \begin{pmatrix} \varepsilon_{3(j-1)+1} I_2 & 0_2 & 0_2 \\ 0_2 & \varepsilon_{3(j-1)+2} I_2 & 0_2 \\ 0_2 & 0_2 & \varepsilon_{3(j-1)+3} I_2 \end{pmatrix}. \tag{S23}$$

with $j \in \{1,2,3,4,5\}$. For the directional coupler, we write

$$C = \begin{pmatrix} \sqrt{1-\beta}\, I_2 & \sqrt{\beta}\, I_2 & 0_2 \\ -\sqrt{\beta}\, I_2 & \sqrt{1-\beta}\, I_2 & 0_2 \\ 0_2 & 0_2 & I_2 \end{pmatrix}. \tag{S24}$$

We define

$$T = CL_5B_2L_4J_3J_4L_3B_2L_2B_1L_1S_{12}. \tag{S25}$$

The displacement d' after the directional coupler can then be calculated by

$$d' = Td_0. \tag{S26}$$

For the covariance matrix V' of the final state, we find the expression

$$V' = TV_0T^\dagger + A, \tag{S27}$$

where the added matrix A can be analytically expressed as

$$A = CL_5B_2L_4J_4J_3L_3B_2L_2B_1A_1B_1^\dagger L_2^\dagger B_2^\dagger L_3^\dagger J_3^\dagger J_4^\dagger L_4^\dagger B_2^\dagger L_5^\dagger C^\dagger \tag{S28}$$

$$+ CL_5B_2L_4J_4J_3L_3B_2A_2B_2^\dagger L_3^\dagger J_3^\dagger J_4^\dagger L_4^\dagger B_2^\dagger L_5^\dagger C^\dagger \tag{S29}$$

$$+ CL_5B_2L_4J_4J_3A_3J_3^\dagger J_4^\dagger L_4^\dagger B_2^\dagger L_5^\dagger C^\dagger \tag{S30}$$

$$+ CL_5B_2L_4J_4N_3J_4^\dagger L_4^\dagger B_2^\dagger L_5^\dagger C^\dagger \tag{S31}$$

$$+ CL_5B_2L_4N_4L_4^\dagger B_2^\dagger L_5^\dagger C^\dagger \tag{S32}$$

$$+ CL_5B_2A_4B_2^\dagger L_5^\dagger C^\dagger \tag{S33}$$

$$+ CA_5C^\dagger. \tag{S34}$$

The displacement for the reference input state is calculated by

$$d = L_2B_1L_1S_{12}d_0. \tag{S35}$$

We rewrite d' and d_{i} as

$$d' = \begin{pmatrix} d_1' \\ d_2' \\ d_3' \end{pmatrix}, \qquad d = \begin{pmatrix} d_1 \\ d_2 \\ d_3 \end{pmatrix}. \tag{S36}$$

The covariance matrix of the input state is given by

$$V = L_2 B_1 L_1 S_{12} V_0 S_{12}^\dagger L_1^\dagger B_1^\dagger L_2^\dagger + A_\mathrm{i} \tag{S37}$$

with

$$A_\mathrm{i} = L_2 B_1 A_1 B_1^\dagger L_2^\dagger + A_2. \tag{S38}$$

We rewrite the resulting covariance matrices in block form

$$V' = \left(\begin{array}{c|c|c} V_1' & * & * \\ \hline * & V_2' & * \\ \hline * & * & V_3' \end{array}\right) \qquad V = \left(\begin{array}{c|c|c} V_1 & * & * \\ \hline * & V_2 & * \\ \hline * & * & V_3 \end{array}\right), \tag{S39}$$

where V_j' denotes the final covariance matrix in path j and V_j denotes the respective input covariance matrix . In the next step, we calculate the Uhlmann-fidelity between the states (d_3, V_3) and (d_1', V_1'), which can be expressed for single mode Gaussian states as

$$F(d_3, V_3, d_1', V_1') = \frac{1}{2} \frac{\exp(-\boldsymbol{\beta}^\mathrm{T}(V_3 + V_1')^{-1}\boldsymbol{\beta})}{\sqrt{\Lambda + \Delta} - \sqrt{\Delta}}, \tag{S40}$$

where $\Lambda = \det(V_3 + V_1')$, $\Delta = 16(\det V_3 - 1/16)(\det V_1' - 1/16)$ and $\boldsymbol{\beta} = d_3 - d_1'$. To fit the experimental data, as shown in Fig. 3 in the main text, we use a least-square fit, where the JPA noise coefficients χ_1, χ_2, and the environmental temperature T are treated as fit parameters. As a result, we minimize the squared distance between the experimentally determined fidelities $\{F(r_i, G_i)\}$ for squeezing r_i and degenerate gain G_i and the theoretically predicted values $F_\mathrm{t}(\chi_1, \chi_2, T, \{\mathbf{x}_i\})$, where χ_1, χ_2, and T are treated as variables and $\{\mathbf{x}_i\}$ is the parameter set including the JPA 1 and JPA 2 squeezing parameters r_i, the degenerate gain G_i of JPA 3 and JPA 4, the amplification angles γ_i as well as the constant system parameters such as path losses, and coupling strength β. Thus, we fit the measurement data by minimizing the function

$$\mathcal{L}(\chi_1, \chi_2, T, \{\mathbf{x}\}, \{F_i\}) = \sum_i |F_\mathrm{t}(\chi_1, \chi_2, T, \{\mathbf{x}\}) - F(r_i, G_i)|^2. \tag{S41}$$

The parameters used in the numerical model are summarized in Tab. S1. The parameters χ_1, χ_2, and T are extracted from the fit routine and the losses are estimated from the data sheets of the

respective passive microwave components. The loss values ε_3, ε_6, ε_7, ε_{10} and ε_{13} are set to zero since they have been artificially introduced to keep the block matrix structure which simplifies the numerical calculation.

Table S1: Model parameters used for the QT protocol fit in the main article. The loss values ε_i are estimated from individual losses of various components. During the fit we have varied three parameteres, χ_1, χ_2, and T.

χ_1	χ_2	T (mK)	β (dB)	ε_1 (dB)	ε_2 (dB)	ε_3 (dB)
0.0158	0.2271	71.7	-15	0.36	0.35	0
ε_4 (dB)	ε_5	ε_6 (dB)	ε_7 (dB)	ε_8 (dB)	ε_9 (dB)	ε_{10} (dB)
0.5	1.1	0	0	0.64	0.63	0
ε_{11} (dB)	ε_{12} (dB)	ε_{13} (dB)	ε_{14} (dB)	ε_{15} (dB)	f_0 (GHz)	n_{d}
0.84	0.88	0	0.5	0.7	5.435	1.1
μ						
0.98						

Supplementary note 3: Bell detection

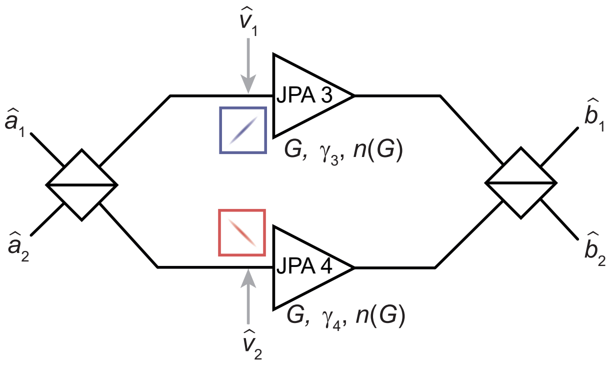

Figure S3: **Josephson mixer scheme.** The frequency-degenerate Josephson mixer consists of JPA 3 and JPA 4 in combination with two symmetric microwave beam splitters to realize the Bell detection.

Alice measures her two-mode Gaussian state with the Josephson mixer setup shown in Figure S3. The projective character of this measurement depends sensitively on the choice of the coupling factor β of the subsequent directional coupler (shown in Fig. S2) and the degenerate gain G of JPA 3 and JPA 4. In the following, we show that the quantity $G\beta$ allows us to distinguish between coherent state quantum teleportation and vacuum teleportation. In the limit $\beta \to 0$, $G \to \infty$, Alice's state detection becomes ideally projective if $G\beta = 4$. Simultaneously, the regime of vacuum teleportation is characterized by $G \to 1$. For $G\beta \gg 4$, the measurement apparatus acts as an amplifier, leading to imperfect interference at the directional coupler. To investigate these regimes, we consider the ideal quantum teleportation protocol (i.e. without losses and noise, no thermal noise) with non-ideal state detection and finite two-mode squeezing. We write the ideal protocol in terms of block matrices. In the following, I_2 denotes the 2×2 identity matrix, $\hat{\sigma}_z$ denotes the Pauli z-matrix, 0_2 is the 2×2 zero matrix and $\mathbf{0} = (0, 0)^{\mathrm{T}}$. The covariance matrix V and the displacement d of the tripartite input state can then be expressed

as

$$V = \frac{1}{4}\begin{pmatrix} I_2 \cosh 2r & \hat{\sigma}_z \sinh 2r & 0_2 \\ \hat{\sigma}_z \sinh 2r & I_2 \cosh 2r & 0_2 \\ 0_2 & 0_2 & I_2 \end{pmatrix}, \qquad d = \begin{pmatrix} \mathbf{0} \\ \mathbf{0} \\ d_\mathrm{i} \end{pmatrix}. \tag{S42}$$

The ideal 50:50 beam splitters, acting on Alice's side, are described by

$$B = \frac{1}{\sqrt{2}}\begin{pmatrix} \sqrt{2}\, I_2 & 0_2 & 0_2 \\ 0_2 & I_2 & I_2 \\ 0_2 & -I_2 & I_2 \end{pmatrix}. \tag{S43}$$

To model the phase-sensitive amplification of JPA 3 and JPA 4, we define the matrices

$$J_3 = \begin{pmatrix} 1/\sqrt{G} & 0 \\ 0 & \sqrt{G} \end{pmatrix} \qquad J_4 = \begin{pmatrix} \sqrt{G} & 0 \\ 0 & 1/\sqrt{G} \end{pmatrix} \tag{S44}$$

and

$$J = \begin{pmatrix} I_2 & 0_2 & 0_2 \\ 0_2 & J_3 & 0_2 \\ 0_2 & 0_2 & J_4 \end{pmatrix}. \tag{S45}$$

For the directional coupler, we write

$$C = \begin{pmatrix} \sqrt{1-\beta}\, I_2 & \sqrt{\beta}\, I_2 & 0_2 \\ -\sqrt{\beta}\, I_2 & \sqrt{1-\beta}\, I_2 & 0_2 \\ 0_2 & 0_2 & I_2 \end{pmatrix}. \tag{S46}$$

As a result, the ideal teleportation protocol can be expressed as

$$T = CBJB = \frac{1}{2}\begin{pmatrix} 2\sqrt{1-\beta}\, I_2 & \sqrt{\beta}(J_3 - J_4) & \sqrt{\beta}(J_3 + J_4) \\ -\sqrt{\beta}\, I_2 & \sqrt{1-\beta}(J_3 - J_4) & \sqrt{1-\beta}(J_3 + J_4) \\ 0_2 & -J_3 - J_4 & -J_3 + J_4 \end{pmatrix}. \tag{S47}$$

The final covariance matrix according to Eq. S47 is calculated by

$$V' = TVT^\dagger. \tag{S48}$$

After some lengthy calculations based on the aforementioned formalism, one can find the local covariance matrix V_f in path 1, corresponding to the output state of the directional coupler

$$V_\mathrm{f} = \frac{1}{16}\Big\{ 4\cosh 2r I_2 + 2\sqrt{\beta}\sinh 2r (J_3 - J_4)\hat{\sigma}_z + \\ + 2\sqrt{\beta}\sinh 2r \hat{\sigma}_z (J_3 - J_4) + \beta\cosh 2r (J_3 - J_4)^2 + \beta (J_3 + J_4)^2 \Big\} \tag{S49}$$

If we now let

$$\beta \to 0, \qquad G \to \infty, \qquad G\beta = 4, \tag{S50}$$

we find that J_3 and J_4, combined with the coupling β, are transformed into the quadrature projection operators

$$\frac{\sqrt{\beta}}{2} J_3 \to \Pi_p = \begin{pmatrix} 0 & 0 \\ 0 & 1 \end{pmatrix}, \qquad \frac{\sqrt{\beta}}{2} J_4 \to \Pi_q = \begin{pmatrix} 1 & 0 \\ 0 & 0 \end{pmatrix}, \tag{S51}$$

where Π_p (Π_q) describes a projective measurement of the p (q) quadrature in the quadrature basis (p, q). The beam splitter between the measurement JPAs and the directional coupler transforms the quadrature measurement basis (p, q) to the hybridized measurement basis $(\tilde{p}, \tilde{q})$, where

$$\tilde{p}(p, q) = \frac{p + q}{2} \tag{S52}$$

denotes the symmetric hybridization and

$$\tilde{q}(p, q) = \frac{p - q}{2} \tag{S53}$$

denotes the antisymmetric hybridization. The terms symmetric and antisymmetric refer to the fact that $\tilde{p}(p, q) = \tilde{p}(q, p)$ and $\tilde{q}(p, q) = -\tilde{q}(q, p)$, i.e. $\tilde{q}(p, q)$ changes its sign when JPA 3 and JPA 4 are exchanged. Geometrically, this operation rotates the phase space by $\pi/4$. The qubit analogue of this operation would be a basis change from z to x via a $\pi/2$-pulse. The measurement operator Π_+ (Π_-) of the symmetric (antisymmetric) hybrid can be obtained with the beam splitter operation

$$\begin{pmatrix} \Pi_+ \\ \Pi_- \end{pmatrix} = \frac{1}{2} \begin{pmatrix} I_2 & I_2 \\ -I_2 & I_2 \end{pmatrix} \begin{pmatrix} \Pi_q \\ \Pi_p \end{pmatrix}, \tag{S54}$$

implying

$$\Pi_+ = \frac{\Pi_p + \Pi_q}{2}, \qquad \Pi_- = \frac{\Pi_p - \Pi_q}{2}. \tag{S55}$$

We note that $\Pi_+ = I_2/2$ and $\Pi_- = -\hat{\sigma}_z/2$, however, we keep the notation in terms of measurement operators to maintain intuition. Under condition Eq. S50, we find for Eq. S49

$$V_\mathrm{f} = \frac{1}{4}\bigg\{ \cosh 2r I_2 + 4\sinh 2r \Pi_- \hat{\sigma}_z + 4\cosh 2r \Pi_-^2 + 4\Pi_+^2 \bigg\}. \tag{S56}$$

As a result, the local covariance matrix V_f of the final teleported state is given by

$$V_\mathrm{f} = \frac{1}{4}(2e^{-2r} + 1)I_2. \tag{S57}$$

Furthermore, the final displacement in path 1 coincides with the displacement of the input state, $d_\mathrm{f} = d_\mathrm{i}$. As a result, the teleportation fidelity F can be expressed by the well-known result

$$F = \frac{1}{\sqrt{\det((e^{-2r}+1)I_2)}} = \frac{1}{1+e^{-2r}}, \tag{S58}$$

which reaches $1/2$ in the classical case $r = 0$. In this case, we have $V_\mathrm{f} = 3I_2/4$, since we add the vacuum fluctuations from path 3 to path 1. In the following, we investigate the effect of finite squeezing and suboptimal measurement gain. In this case, we reconsider the final covariance matrix and calculate the displacement without the assumption that the projection conditions Eq. S50 hold

$$\begin{aligned} V_\mathrm{f} = \frac{I_2}{16}\bigg\{ \cosh 2r \bigg[4(1-\beta) + \beta\left(\sqrt{G} - \frac{1}{\sqrt{G}}\right)^2\bigg] - \\ -4\sqrt{\beta(1-\beta)}\left(\sqrt{G} - \frac{1}{\sqrt{G}}\right)\sinh 2r + \beta\left(\sqrt{G} + \frac{1}{\sqrt{G}}\right)^2 \bigg\}. \end{aligned} \tag{S59}$$

$$d_\mathrm{f} = \frac{\sqrt{\beta}}{2}\left(\sqrt{G} + \frac{1}{\sqrt{G}}\right) d_\mathrm{i}. \tag{S60}$$

We consider the classical case, hence

$$V_\mathrm{f} = \frac{I_2}{8}\left(2(1-\beta) + \beta G + \frac{\beta}{G}\right). \tag{S61}$$

From that, we obtain the result

$$F_\mathrm{c} = \frac{\exp\left(-|\alpha|^2 \frac{B(\beta,G)}{A(\beta,G)}\right)}{2A(\beta,G)}, \tag{S62}$$

with

$$A(\beta, G) = \frac{1}{8}\left(4 - 2\beta + \beta G + \frac{\beta}{G}\right), \tag{S63}$$

$$B(\beta, G) = \left(\frac{\sqrt{\beta}}{2}\left(\sqrt{G} + \frac{1}{\sqrt{G}}\right) - 1\right)^2. \tag{S64}$$

We first consider the case without displacement, $|\alpha|^2 = 0$. With $\beta \ll 1$, we obtain

$$F_{\mathrm{c}}(G, \beta) = \frac{4}{4 - 2\beta + \beta G + \beta/G}. \tag{S65}$$

We observe that $F(1, \beta) = 1$, as expected for vacuum teleportation. If we meet the projection condition $G\beta = 4$, we obtain the expected threshold $F = 1/2$ for $G \to \infty$, $\beta \to 0$. For finite β and $G \to \infty$, we obtain $F \to 0$ since we strongly amplify Alice's part of the TMS (which locally looks like a thermal state) and hence cannot achieve destructive interference at the directional coupler. We assume that $\beta \ll 1$ and $G \gg 1$ and define the quantity $k = \beta G/4$. This quantity describes the "projectiveness" of the measurement. In the quantum case, $r \neq 0$, we then find the fidelity

$$F_{\mathrm{q}} = \frac{\exp\left(-|\alpha|^2 \frac{(\sqrt{k}-1)^2}{C(r,k)}\right)}{C(r, k)}, \tag{S66}$$

where we define the function

$$C(r, k) = (1 + k)\cosh^2 r - \sqrt{k}\sinh 2r. \tag{S67}$$

The classical fidelity can be rewritten as

$$F_{\mathrm{c}} = \frac{\exp\left(-|\alpha|^2 \frac{(\sqrt{k}-1)^2}{1+k}\right)}{1 + k}. \tag{S68}$$

Supplementary note 4: Gaussianity Verification

Our quantum state tomography relies on the assumption that reconstructed quantum states are Gaussian. In this way, a complete description of the state by first and second order moments

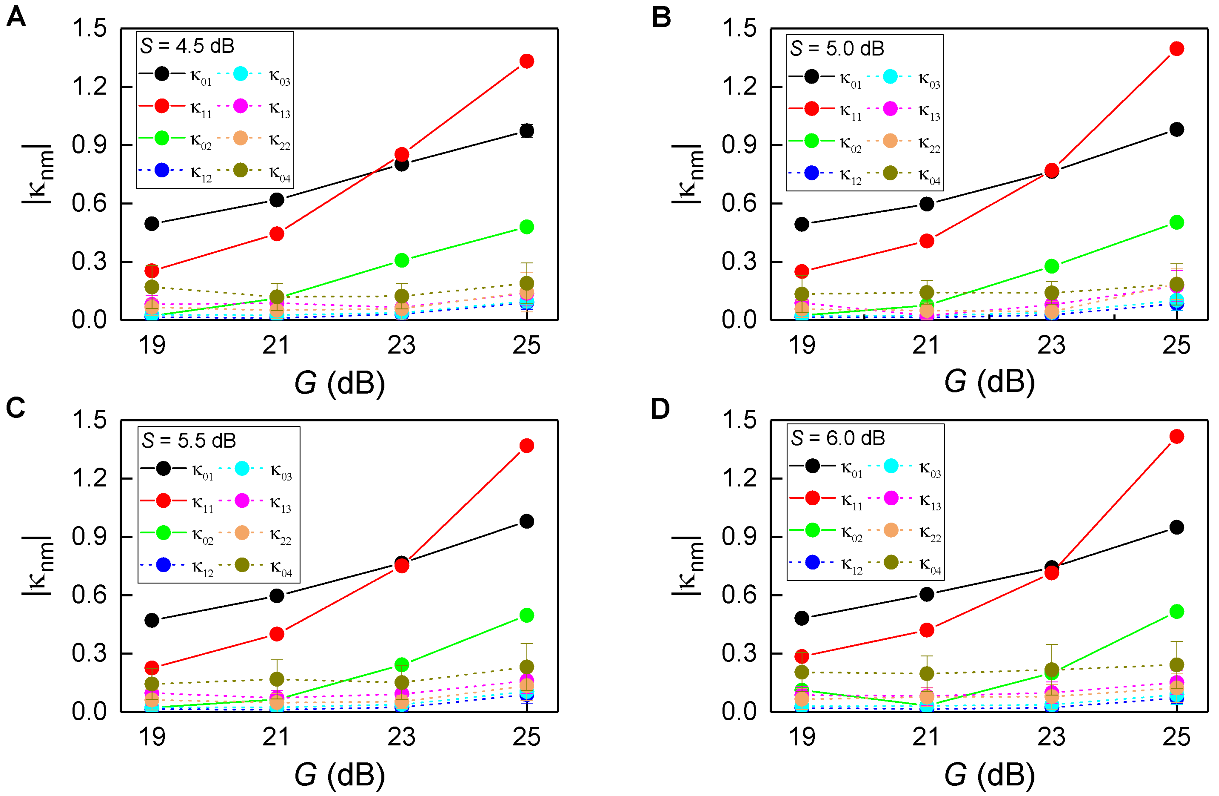

Figure S4: **Cumulants of teleported coherent states.** Absolute values of cumulants $|\kappa_{nm}|$ as a function of the measurement gain G for the squeezing levels (**A**) $S = 4.5\,\mathrm{dB}$, (**B**) $S = 5.0\,\mathrm{dB}$, (**C**) $S = 5.5\,\mathrm{dB}$, and (**D**) $S = 6.0\,\mathrm{dB}$. Lines are guides for the eyes. Error bars reflect the respective standard deviations. If not shown, error bars are smaller than the symbol size.

of bosonic field operators is possible. This assumption of Gaussianity can be justified by analyzing the cumulants κ_{nm} of order $n+m$, where n and m are positive integers, which are given by

$$\kappa_{nm} = \frac{\partial^n}{\partial x^n}\frac{\partial^m}{\partial y^m} \ln \sum_{\alpha,\beta} \frac{\langle(\hat{a}^\dagger)^\alpha \hat{a}^\beta\rangle x^\alpha y^\beta}{\alpha!\beta!}\Bigg|_{x=y=0}, \tag{S69}$$

where $\langle(\hat{a}^\dagger)^\alpha \hat{a}^\beta\rangle$ are the signal moments. Gaussian states are characterized by $\kappa_{nm} = 0$ for $n+m > 2$. In Fig. S4, we plot absolute values of the experimentally determined cumulants κ_{01}, κ_{11}, κ_{02}, κ_{12}, κ_{03}, κ_{22}, κ_{04} and κ_{13} for the teleported states shown in Fig. 3 a, b in the main text. We observe that $|\kappa_{01}|, |\kappa_{11}| \gg |\kappa_{nm}|$ for $3 \leq n+m \leq 4$ and $|\kappa_{02}| \gg \kappa_{nm}$ for $G \geq 23\,\mathrm{dB}$.

Since only the cumulants up to second order are significantly different from zero, it is justified to treat the teleported states as Gaussian ones.

Supplementary note 5: Effect of Imperfections on Teleportation Fidelity

Detailed investigation of an error budget of microwave quantum teleportation is a very complicated task due to the highly nonlinear nature of teleportation fidelity as a function of losses, noise, and other imperfections. However, most important and general sources of infidelity can be identified by using the developed theory model. Figure S5 illustrates the impact of two most strongest sources of imperfections on the maximum fidelity of microwave quantum teleportation protocol. The maximum fidelity is defined as $F_{\max}(S) = \max(F(G, S = \text{const}))$. As a baseline we use the default parameter set which has been obtained by fitting the experimental data with our theory model (see Fig. 3). Next, we consider three particular scenarios: (i) ten-fold reduced losses $\varepsilon_i \rightarrow \varepsilon_i/10$, (ii) ten-fold reduced noise parameters in JPAs $\chi_i \rightarrow \chi_i/10$, and (iii) combination of both. The corresponding results are shown in Fig. S5. First of all, one can notice that a small improvement in terms of fidelity should be possible even with the current experimental parameters by slightly increasing the squeezing level S, since the black line maximum in Fig. S5 lies slightly outside of the experimentally studied range of the squeezing levels. Next, one can observe that the impact of the transmission losses reduction is greater than the impact from the noise reduction. Technologically, this is an important finding, since transmission losses are often more straightforward to improve.

One particular source of transmission losses is due to finite insertion losses in the current cryogenic hybrid rings. These losses are around 0.4 dB per hybrid ring and unavoidably reduce the amount of quantum correlations, couple extra thermal noise, and therefore, reduce the purity and squeezing of the propagating TMS signals. Based on the predictions of our theory model, one can roughly estimate that the individual hybrid ring insertion losses account for ap-

proximately $2-4\,\%$ of the teleported state infidelities in our experiment (assuming the optimal measurement gain G and squeezing level S). In the end, an obvious improvement of the current experimental set-up would be to use superconducting hybrid rings with negligible insertion losses, which could potentially improve the current teleportation fidelities by an extra $6-10\,\%$

Optimization of JPA noise is a far more subtle and complicated task. Ultimately, the improvement of both losses and JPA noise properties is required in order to reach fidelites beyond $90\,\%$, as illustrated by cyan line in Fig. S5.

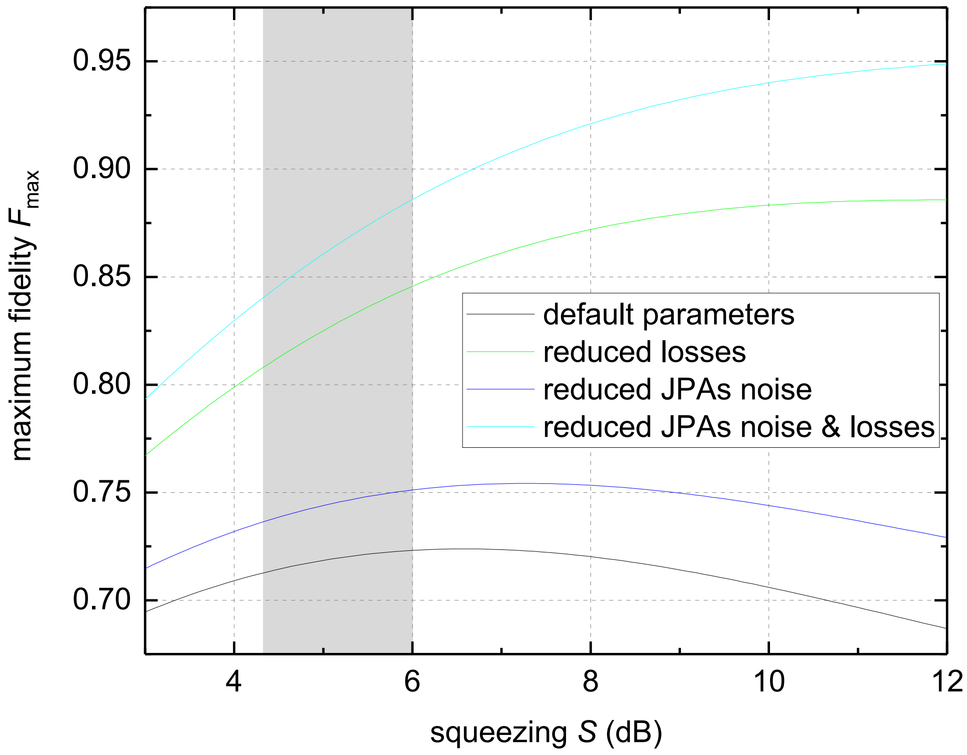

Figure S5: **Effect of imperfections in microwave teleportation.** Here, we predict the maximum teleportation fidelity as a function of the initial squeezing level S for four different sets of theory model parameters. Default parameters correspond to the set of theory parameters used in Fig. 3 for fitting of the experimental data and serve as a reference. Reduced losses correspond to the ten-fold flat reduction of all transmission losses $\varepsilon_i \to \varepsilon_i/10$. Reduced JPA noise corresponds to the ten-fold reduction in terms of noise parameters $\chi_i \to \chi_i/10$. Grey area highlights the range of the squeezing levels studied in the current experiment.
